# Impact of hypothetical improvements in the psychosocial work environment on sickness absence rates: a simulation study

**DOI:** 10.1093/eurpub/ckac109

**Published:** 2022-08-27

**Authors:** Jimmi Mathisen, Tri-Long Nguyen, Johan H Jensen, Amar J Mehta, Reiner Rugulies, Naja H Rod

**Affiliations:** Section of Epidemiology, Department of Public Health, University of Copenhagen, Copenhagen, Denmark; Copenhagen Stress Research Center, Copenhagen, Denmark; Section of Epidemiology, Department of Public Health, University of Copenhagen, Copenhagen, Denmark; Copenhagen Stress Research Center, Copenhagen, Denmark; Department of Occupational and Environmental Medicine, Bispebjerg and Frederiksberg Hospital, University of Copenhagen, Copenhagen, Denmark; Section of Epidemiology, Department of Public Health, University of Copenhagen, Copenhagen, Denmark; Copenhagen Stress Research Center, Copenhagen, Denmark; Section of Epidemiology, Department of Public Health, University of Copenhagen, Copenhagen, Denmark; Copenhagen Stress Research Center, Copenhagen, Denmark; National Research Centre for the Working Environment, Copenhagen, Denmark; Department of Psychology, University of Copenhagen, Copenhagen, Denmark; Section of Epidemiology, Department of Public Health, University of Copenhagen, Copenhagen, Denmark

## Abstract

**Background:**

The association between psychosocial working environments and sickness absence is well-known. However, the potential for reducing sickness absences of different lengths through improvements in psychosocial work factors is not fully understood. We aim to quantify the potential for reducing short-, intermediate- and long-term sickness absence rates, respectively, through hypothetical improvements in several psychosocial work factors.

**Methods:**

This longitudinal study includes 24 990 public hospital employees from the 2014 wave of the *Well-being in Hospital Employees* study. The 1-year sickness absence rate was divided into short- (1–3 days), intermediate- (4–28 days) and long-term (29 days or more) periods. We simulated hypothetical scenarios with improvements in 17 psychosocial work factors using the parametric g-formula and estimated resulting changes in sickness absence rate ratios (RRs) with 95% confidence intervals (95% CIs).

**Results:**

Setting all 17 psychosocial work factors to their most desirable levels (vs. least desirable levels) was associated with an overall 54% lower rate of sickness absence (95% CI: 48–60%). Reducing bullying (no vs. yes RR: 0.86, 95% CI: 0.83–0.90) and perceived stress (low vs. high RR: 0.90, 95% CI: 0.87–0.92), and increasing skill discretion (high vs. low RR: 0.91, 95% CI: 0.89–0.94) held the largest potential for reducing the total sickness absence rate. Overall, associations were similar for short-, intermediate- and long-term sickness absence.

**Conclusions:**

The psychosocial working environment was strongly associated with sickness absence. Improving the working environment may have a great impact on short-, intermediate- and long-term sickness absence rates.

## Introduction

Sickness absence often indicates impaired health and functioning in individual employees,^1–3^ and high sickness absence rates are costly for employers and societies.^4^ It is therefore important to identify relevant targets for intervention. A wide variety of exposures in the psychosocial working environment, such as bullying, low leadership quality and low influence at work, have been associated with sickness absence^5–9^ and thus, improvements in the psychosocial working environment hold promise for reducing sickness absence at the workplace level.

Short-term absence often represents a substantial proportion of total sickness absence rates,^10^ yet most studies on the association between psychosocial work environments and sickness absence do not specifically include short-term absences. The psychosocial working environment may influence both shorter and longer spells of sickness absence, but not necessarily through similar pathways.^11–13^ For example, Thorsen *et al*.^11^^,^^13^ reported that perceived stress was more strongly associated with long-term sickness absence than with overall sickness absence among women and that poor influence was more strongly associated with overall sickness absence than long-term sickness absence among men. Another study by Nielsen *et al*.^12^ reported that psychological demands were associated with long-term absence, but not short-term absence among women. To reduce both shorter- and longer-term sickness absence rates, it is, therefore, important to investigate how improving the psychosocial work environment would affect sickness absence spells of different lengths.

Evidence of the impact of improvements or interventions should ideally come from trials. However, it may be difficult to conduct trials on psychosocial work factors, for both ethical and feasibility reasons. As an alternative, observational data can be used to simulate hypothetical improvements and interventions via the parametric g-formula.^14–16^ This tool can be used to simulate contrasts between hypothetical exposure scenarios and estimate the resulting changes in a population-level outcome. In our setting we could, for example, contrast a simulated scenario where everyone were exposed to high leadership quality at the workplace with an actual scenario where everyone were exposed to their reported level of leadership quality. The possible discrepancy in the predicted sickness absence rates under these two scenarios would provide an estimate of the potential impact of improving leadership quality on sickness absence.

The objective of this study is to simulate hypothetical improvements in a wide variety of psychosocial work factors and estimate the association with sickness absence in a large cohort of hospital employees. We aim to quantify the overall preventive potential associated with an optimal psychosocial working environment, as well as identify which single factors hold the greatest potential for reducing total, short-, intermediate- and long-term sickness absence rates.

## Methods

### Study population

**Figure 1:**
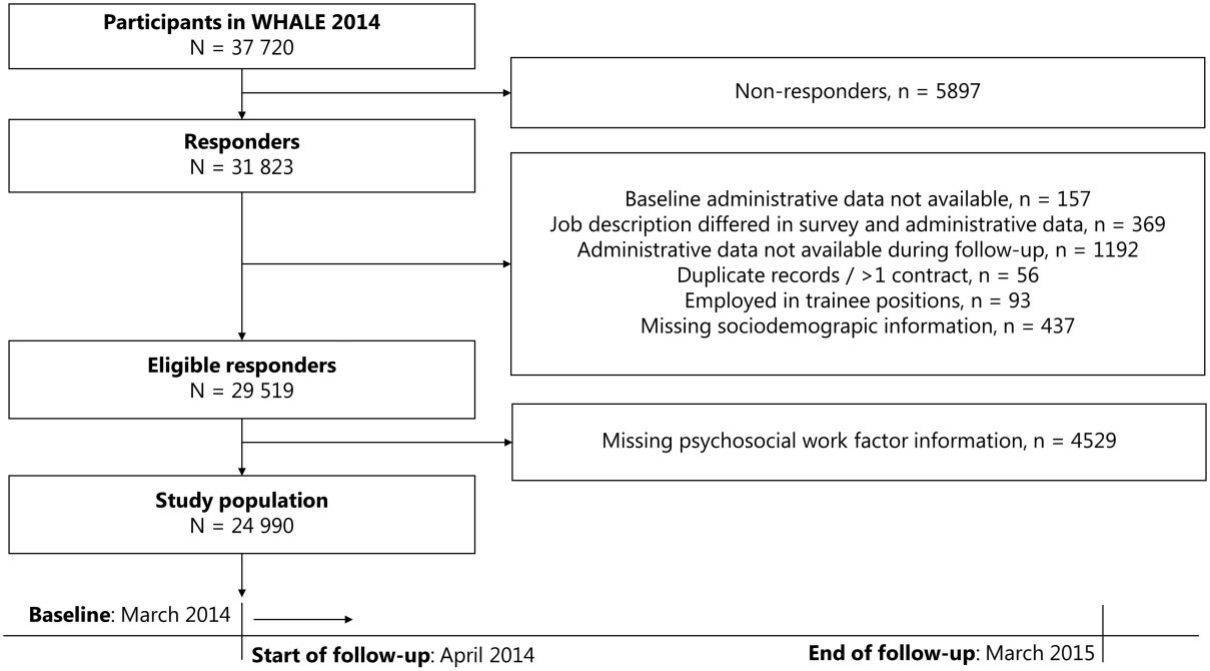
Flow chart of the study population and illustration of the follow-up period

The study population consisted of participants in the 2014 wave of the *Well-being in Hospital Employees* study (WHALE),^17^ including all employees in the public healthcare enterprise in the Capital Region of Denmark in March 2014. Data included self-reported workplace survey assessments, monthly updated employer-based administrative registers and Danish national registries. Among the 37 720 employees invited to the survey, 31 823 (84%) responded (figure 1). We excluded individuals who had a lack of/errors in employer-based administrative data (*n* = 1718), were employed in multiple or trainee positions (*n* = 149) or had missing sociodemographic information (*n* = 437). From the eligible responders (*n* = 29 519), we included only employees with full information on psychosocial work factors (*n* = 24 990) in the study population. To reduce potential selection bias due to missing information we applied inverse probability weighting to allow the study sample to recover information on employees with incomplete data on psychosocial work factors.^18^

### Psychosocial work factors

The WHALE data contain 40 self-reported items on psychosocial work factors, mostly measured on 5- or 7-point Likert scales. The majority were similar to items from the Copenhagen Psychosocial Questionnaire II (COPSOQ II).^19^ Where possible, we collapsed items into scales similar to those used in COPSOQ-II. The scales were created by converting the individual item Likert values to a score between 0 and 100 and then averaging the score across all items included in the scale.

We grouped the psychosocial work factors into *psychosocial working conditions* and *cognitive and emotional reactions* to these conditions, drawing inspiration from a framework for research in psychosocial work environments and health.^20^ We included the following psychosocial working conditions: Bullying; Collaboration; Inclusiveness; Influence on work; Influence on schedule; Justice; Leadership quality; Predictability; Role clarity; Sexual harassment; Skill discretion; Trust; Threats; Violence; and Work demands. Furthermore, we included the following cognitive and emotional reactions: Job satisfaction and Perceived stress.

Most scale and individual item values were categorized into *low*, *medium* and *high*, by the 25th and 75th percentile, or as close as possible. Exceptions were items regarding exposure to bullying, sexual harassment, threats and violence within the past 12 months, which were dichotomized (*yes*/*no*). We defined the *most desirable level* of these work factors as ‘High’ (i.e. the upper 25% quartile) for all factors except for bullying, sexual harassment, threats and violence, which were set to ‘No’. We defined the least desirable levels as the reversed levels [*Low* (i.e. the lower 25% quartile); *Yes*]. Further details are available in Supplementary material S1.

### Other covariates

We used information regarding the sociodemographic characteristics of the employees including age, sex, occupational group, household income and marital status. We also used information on the employees’ workplace and employment characteristics including part-time/full-time employment status, seniority and workplace. All information was derived from employer-based administrative data, except information on household income and marital status, which was drawn from national registries. All information was ascertained at baseline (March 2013).

### Sickness absence

Sickness absence was ascertained between April 2014 and March 2015 in employer-based administrative registries. We calculated the total rate of sickness absence by dividing the number of registered sickness absence hours by the total number of fixed working hours during the 1-year follow-up period. We calculated short-term (1–3 days), intermediate-term (4–28 days) or long-term (29 days or more) specific rates by dividing term-specific sickness absence hours by the total number of fixed working hours.

### Analytical framework

We estimated changes in rates of sickness absence under simulated hypothetical exposure scenarios using the parametric g-formula.^15^^,^^16^ Two types of rate ratios (RRs) (denoted *contrasts*) and 95% confidence intervals (CIs) were estimated. First, to estimate overall associations between psychosocial work factors and sickness absence, we calculated *etiologic contrasts.* Here, we compared predicted sickness absence rates in exposure scenarios where all employees were simulated to have the most desirable levels of psychosocial work factors with scenarios where everyone were simulated to have the least desirable levels. Etiologic contrasts correspond to effect estimates from a randomized controlled trial comparing an ‘all-treated’ group with a ‘none-treated’ group and are independent of the distribution of the psychosocial work factors in the study population.^21^ Second, to estimate associations between improvements in psychosocial work factors and sickness absence in this specific cohort, we calculated *realistic contrasts.* Here, we compared predicted sickness absence rates in scenarios where all employees were simulated to have the most desirable levels of psychosocial work factors to scenarios where everyone had their observed levels. As the latter scenario corresponds to the actual distribution of psychosocial work factors in the study population, realistic contrasts mimics effect estimates from an intervention that removes an exposure from a real-life setting, similar to population attributable fractions.^15^^,^^21^ For both contrast types, we simulated improvements in each psychosocial work factor separately as well as in all factors simultaneously. We estimated contrasts separately for total, short-, intermediate- and long-term sickness absence. The g-formula method shares assumptions (such as no unmeasured confounders) with other methods for analysing observational data.

Predicted sickness absence rates were derived from Poisson regression models fitted to predict the individual number of sickness absence hours. The total number of fixed work hours during follow-up was used as an offset in the models to accommodate that the number of work hours under risk differed between employees (due to, for example, employees being employed part-time or only during some of the follow-up period). To avoid adjusting for potential mediators, we fitted two sets of regression models: one set predicting sickness absence from psychosocial working conditions conditional on sociodemographic factors and workplace and employment characteristics, and another set predicting sickness absence from cognitive and emotional reactions conditional on all covariates included in the first set. In total, we fitted eight regression models. We fitted regression models separately for each outcome (total, short-, intermediate- and long-term sickness absence). All models were weighted by inverse probability weights to account for missingness on psychosocial work factors. The weights were constructed by estimating the inverse probability of having incomplete information on psychosocial work factors among all eligible responders conditional on sociodemographic factors and workplace and employment characteristics. All models were fitted using ridge regression to avoid overfitting because of, for example, multicollinearity between psychosocial work factors.^22^ To quantify estimation uncertainty and estimate 95% CIs, we applied bootstrapping via the *Bag of Little Bootstraps* algorithm.^23^

We conducted four sensitivity analyses. First, as previous studies have found sex-specific differences in the associations between psychosocial work factors and sickness absence of different lengths,^11–13^ we estimated associations in women and men separately. Second, sickness absence rates vary greatly between occupational groups within the healthcare sector.^24^^,^^25^ Therefore, we estimated associations between hypothetical improvements in all work factors simultaneously and total sickness absence in each occupational group. Third, prior sickness absence may affect the employees’ perception of their psychosocial work environment. Hence, we adjusted the main analyses for binary variables indicating whether employees had (or had not) had short-, intermediate- or long-term sickness absences in the year before the start of follow-up (April 2013–March 2014). Fourth, we investigated whether our approach of dividing sickness absence rates by their length into three distinct outcomes could bias the estimates due to conditioning on prior and future events. Elaboration of this analysis is presented in Supplementary material S2.

All analyses were conducted in SAS 9.4 and R 4.1.1.^26^^,^^27^

## Results

The mean individual total sickness absence rate was 4.0% (table 1). Of the total rate, short-term sickness absence accounted for 37%, intermediate-term accounted for 32% and long-term accounted for 31%. Physicians and administrative leaders had the lowest sickness absence rates while social and health care employees had the highest rate. The distributions of sickness absence among nurses, physicians and other health care employees leaned towards shorter absences, while the distribution among social and health care employees and service and technical employees leaned towards longer absences. In general, employees reporting more favourable psychosocial working conditions and cognitive and emotional reactions had lower sickness absence rates of all lengths (Supplementary material S3, table S1).

**Table 1:** Descriptive characteristics of the study population and total, short-, intermediate- and long-term sickness absence rates

	*N*	% of *N*	% totalsickness absence	% short-term sickness absence	% intermediate-term sickness absence	% long-term sickness absence
**Total**	24 990	100	4.0	1.5	1.3	1.2
**Sociodemographic factors**						
**Age, years^a^**						
18–34	4797	19	4.0	1.8	1.2	1.0
35–44	6762	27	3.9	1.6	1.2	1.1
45–54	7117	28	3.8	1.4	1.3	1.2
55–64	5651	23	4.3	1.3	1.4	1.6
65 or older	663	3	4.1	1.1	1.1	1.9
**Sex**						
Women	19 674	79	4.3	1.6	1.3	1.3
Men	5316	21	3.0	1.1	1.0	0.8
**Marital status**						
Unmarried	10 959	44	4.5	1.7	1.4	1.4
Married	14 031	56	3.6	1.4	1.2	1.1
**Household income, DKK^b^ × 1000^a^**						
Less than 225	5406	22	5.0	1.8	1.6	1.6
225–299	7818	31	4.4	1.6	1.4	1.3
300–374	5460	22	3.7	1.4	1.2	1.1
375 or more	6306	25	2.9	1.1	0.9	0.9
**Occupational group**						
Physicians	2863	11	2.1	0.9	0.5	0.7
Nurses	8593	34	4.2	1.7	1.4	1.1
Social and health care employees	1825	7	5.9	1.7	2.1	2.1
Other health care employees^c^	3888	16	3.9	1.6	1.2	1.1
Pedagogical employees	633	3	5.1	1.6	1.7	1.7
Service and technical employees	2552	10	4.6	1.2	1.7	1.7
Administrative leaders	393	2	1.8	0.7	0.6	0.6
Administrative employees	4243	17	3.9	1.5	1.1	1.3
**Workplace and employment characteristics**						
**Seniority, years^a^**						
Less than 2	4076	16	4.5	1.8	1.4	1.3
2–4	4243	17	3.9	1.5	1.3	1.1
5–9	6947	28	4.1	1.5	1.3	1.3
10–14	3449	14	3.7	1.4	1.2	1.1
15–24	3330	13	3.8	1.3	1.2	1.2
25 or more	2945	12	3.7	1.2	1.3	1.2
**Part/full-time work^d^**						
Part-time	8695	35	4.9	1.7	1.1	1.0
Full-time	16 295	65	3.5	1.4	1.1	1.0

a Categorized only for descriptive purposes; they are handled as continuous covariates in the regression models.

b DKK = Danish Kroner.

c ‘Other healthcare employees’ include physiotherapists, midwives, biomedical laboratory employees, occupational therapists and radiographers.

d Full-time employment is defined as 37 h per week or more. Part-time employment is defined as <37 h per week.

**Figure 2:**
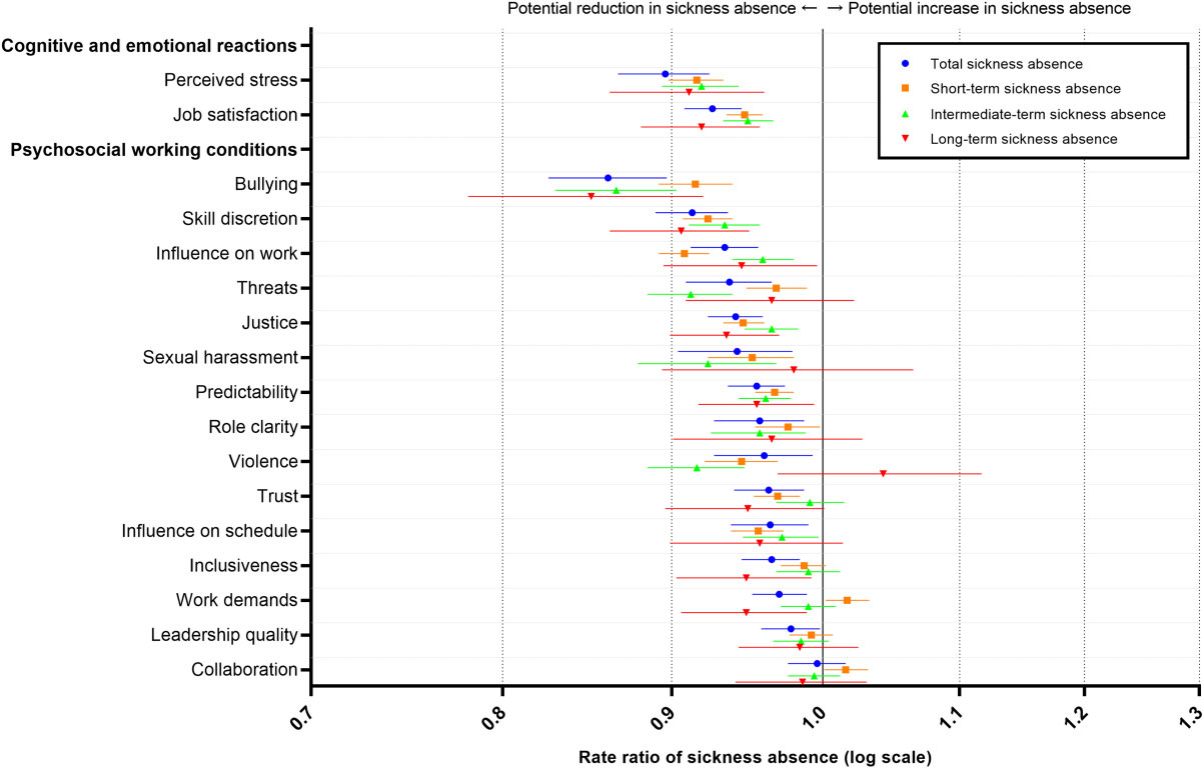
Rate ratios and 95% confidence intervals of sickness absence in the ‘etiologic contrast scenarios’ (most desirable vs. least desirable levels of psychosocial work factors). All estimates are reported in Supplementary material S3 (table S2)

To estimate overall associations between psychosocial work factors and sickness absence of different lengths, we calculated etiologic contrasts (most desirable levels compared with least desirable levels) for all factors simultaneously. The RRs were 0.46 (95% CI: 0.40–0.52) for total sickness absence, 0.59 (95% CI: 0.54–0.63) for short-term sickness absence, 0.54 (95% CI: 0.48–0.60) for intermediate-term sickness absence and 0.47 (95% CI: 0.34–0.61) for long-term sickness absence (Supplementary table S2). For the individual factors, the total sickness absence RRs ranged from 0.86 (95% CI: 0.83–0.90) to 1.00 (95% CI: 0.98–1.02) (figure 2, Supplementary table S2). The strongest associations were found for bullying (RR: 0.86, 95% CI: 0.83–0.90), perceived stress (RR: 0.90, 95% CI: 0.87–0.92) and skill discretion (RR: 0.91, 95% CI: 0.89–0.94). There were only slight variations in the magnitude of the associations across different lengths of sickness absence for each factor.

**Figure 3:**
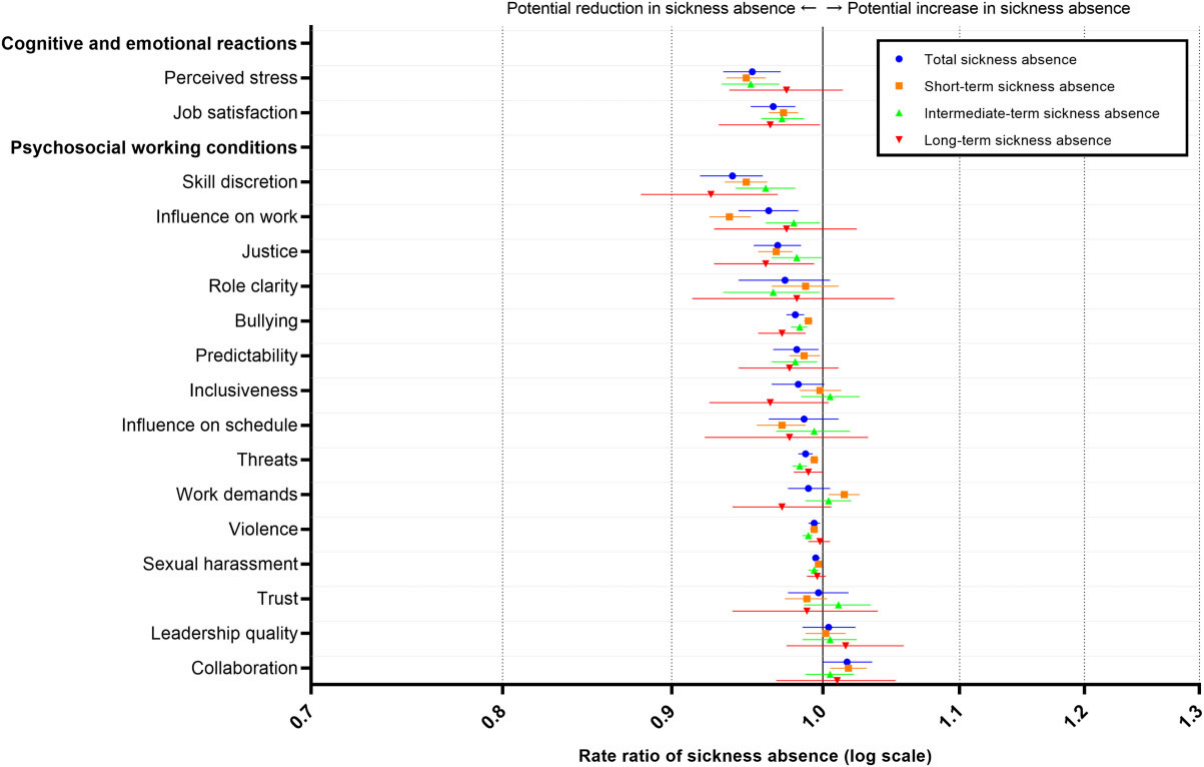
Rate ratios and 95% confidence intervals of sickness absence in the ‘realistic contrast scenarios’ (most desirable vs. observed levels of psychosocial work factors). All estimates are reported in Supplementary material S3 (table S3)

To estimate the potential for reducing sickness absence in this specific cohort, we estimated realistic contrasts (most desirable levels compared with observed levels) for all factors simultaneously. As expected, these associations were weaker than in the etiologic scenario. The RRs were 0.70 (95% CI: 0.64–0.76) for total sickness absence, 0.74 (95% CI: 0.70–0.78) for short-term sickness absence, 0.78 (95% CI: 0.71–0.85) for intermediate-term sickness absence and 0.65 (95% CI: 0.52–0.79) for long-term sickness absence (Supplementary table S3). These RRs correspond to, for example, a reduction in the total sickness absence rate of 30% (95% CI: 24–36%) (Supplementary table S4). For the individual factors, the RRs ranged from 0.94 (95% CI: 0.92–0.96) to 1.02 (95% CI: 1.00–1.03) for total sickness absence (figure 3, Supplementary table S3). The strongest individual associations with total sickness absence were found for skill discretion (RR: 0.94, 95% CI: 0.92–0.96), perceived stress (RR: 0.95, 95% CI: 0.93–0.97) and influence on work (RR: 0.96, 95% CI: 0.94–0.98). Again, there were only small variations across different lengths of sickness absence.

The estimated associations were similar in women and men (Supplementary tables S5 and S6) and across all occupational groups (Supplementary table S7). Furthermore, adjusting for sickness absence in the year preceding baseline attenuated the associations, but the general patterns remained stable (Supplementary table S8). Lastly, accounting for the potential influence of conditioning on prior and future sickness absence events yielded results that were similar to the main analysis (Supplementary table S9).

## Discussion

In this large prospective cohort of public hospital employees, we found strong associations between psychosocial work factors and sickness absence. We estimated a 54% lower sickness absence rate when comparing simulated scenarios with the most vs. the least desirable levels of all factors. Reductions in bullying and perceived stress, and increases in skill discretion seemed to have the largest impact on sickness absence rates. Several other hypothetical improvements, such as increasing influence on work, justice and job satisfaction seemed to have a moderate impact. Furthermore, we estimated that 30% of the sickness absence in this specific cohort could potentially be prevented. Contrary to our hypotheses, we did not find meaningful differences in the associations between psychosocial work factors and sickness absence according to the length of the sickness absence spells. The findings were robust to various sensitivity analyses.

Many psychosocial work factors are associated with sickness absence, and our findings are, therefore, generally in line with previous studies.^5–9^ Specifically, we found that exposure to bullying and perceived stress were among the strongest determinants of sickness absence, and these associations are well-documented.^5^^,^^11^^,^^28^ We also found a strong association between low skill discretion and sickness absence. Low skill discretion correlates with low socioeconomic position, and low socioeconomic position is strongly associated with sickness absence in Denmark.^29^ Thus, confounding by socioeconomic position appears conceivable. However, as we adjusted for two measures of socioeconomic position, occupational position and household income, it seems that low skill discretion contributes to the risk of sickness absence independently of socioeconomic position. In contrast to previous studies, we did not find clear associations between sickness absence and leadership quality^6^^,^^7^^,^^30^ or violence.^5–7^ Leadership quality may influence the risk of sickness absence through several other working conditions, for example, work demands, skill discretion, predictability and influence on work. The simultaneous inclusion of these potentially mediating factors in the analyses could, therefore, explain the lack of an association between leadership quality and sickness absence in this study.

We found that up to 30% of the sickness absence in this population could be prevented through improvements in the psychosocial work environment. This is similar in magnitude to previously reported population attributable fractions (between 29% and 32%, respectively).^6^^,^^31^^,^^32^ Although this potential seems large, most hypothetical improvements in individual factors showed only limited preventive potential. Psychosocial working environments are highly complex, and the constructs used for measuring these are overlapping and intertwined.^33^ Therefore, changes in only one dimension of the working environment are perhaps less likely to elicit a large effect on sickness absence compared with more composite changes.^34^ Future research could simulate more comprehensive interventions in the work environment and quantify their potential for reducing sickness absence rates.

Contrary to our hypotheses, and contrary to previous findings,^11–13^ we did not find meaningful differences in associations between the psychosocial work factors and sickness absence according to the length of sickness absence. There are several possible explanations for this discrepancy. First, clear differences in the analytical approach exist between our study and the previous studies. For example, whereas previous studies reported regression coefficients, we reported predicted RRs from simulated and observed exposure scenarios. Second, the conceptualization of different lengths of sickness absence varies between the studies. For example, Nielsen *et al*.^12^ use 11 consecutive days as the cut-off between short- and long-term absence, while Thorsen *et al*.^11^^,^^13^ do not consider short-term absences separately. Third, whereas previous studies were conducted among employees from a range of occupational sectors, our study included employees from the public hospital sector only.

In a previous study, we used a similar analytical approach to study the potential for preventing employee turnover in the same cohort.^35^ We found that perceived stress, bullying and skill discretion were also important determinants of turnover. The strongest determinant of turnover was job satisfaction, which only had a modest impact on sickness absence in the current study. Some elements of leadership quality were moderately associated with turnover, while it does not seem to have a notable impact on sickness absence in the current study. Thus, improvements in the psychosocial working environment appear to have the potential to reduce both turnover and sickness absence,^36^ but the strongest determinants of each outcome seem to differ in this cohort.

### Strengths and limitations

The strengths of the study include the large and comprehensive cohort with high participation and detailed data. This enabled the estimation of associations between a wide variety of psychosocial work factors and sickness absence rates of different lengths as well as the inclusion of many potential confounders. We used the parametric g-formula to estimate conventional *high* vs. *low exposure* associations but also population-specific *high* vs. *observed exposure* associations, which are arguably more informative for decision-making.^21^ To our knowledge, this analytic approach is novel in this field.

In the main analysis, we treated short-, intermediate- and long-term sickness absence rates as mutually exclusive, yet the true rates likely depend on each other. In a sensitivity analysis, we accommodated this interdependency to some extent, and it did not substantially change the results. However, it can be argued that reducing, for example, long-term sickness absence rates may increase intermediate- or short-term sickness absence rates as some long-term sickness absence episodes are more likely to be shortened than to be eliminated. Accounting for this issue would require, for example, multi-state modelling that allows transitions between different sickness absence lengths,^37^ which was beyond the scope of the study.

We assumed that the psychosocial work factors causally preceded sickness absence. Yet, prior sickness absence may also affect the employees’ perception of their psychosocial work environment. Thus, we conducted a sensitivity analysis in which we adjusted for sickness absence in the year preceding baseline. This only had a minor impact on the results.

Information about the underlying illnesses that caused the sickness absences was unfortunately not available. From the literature, it can be inferred that shorter absences tend to be caused by illnesses with a limited time span, such as colds or influenza, while longer absences tend to be caused by illnesses with a longer time span, such as mental health conditions.^25^ Yet, both may be affected by psychosocial working environment factors. For example, persons who suffer from psychological stress and lack of social support are more prone to develop colds and influenza when exposed to viruses,^38^ while prospective associations have been found between adverse working environments and depression.^39^ It would have been interesting to stratify our analyses by cause of sickness absence as this would have provided information about whether psychosocial work environment factors were to a greater or lesser extent associated with diagnosis-specific sickness absence spells.

### From simulated improvements to real interventions

We estimated a large potential for reducing sickness absence through hypothetical improvements in the psychosocial working environment. This estimate reflects an upper bound of the potential for reducing sickness absence through improvements in the psychosocial working environment rather than a realistic target in a real-life setting.

Transferring the knowledge from this study into actual interventions will require careful consideration and further research. Consider, for example, perceived stress, which was one of the factors with the largest potential for reducing sickness absence. Although there is evidence that targeting the experience of stress on the individual level through, for example, cognitive behaviour therapy is beneficial for employees, evidence for an effect on preventing sickness absence is weak.^40^ Moreover, interventions in individuals do not tackle the underlying environmental causes of stress such as, for example, stressful working conditions. In theory, interventions targeting organizational parameters (e.g. the working conditions) should be more effective than individual-level interventions, yet the evidence regarding their effectiveness is weak.^34^ Similar considerations about intervention design and implementation will also apply to the other psychosocial work factors measured in this study.

## Conclusions

In summary, we found that the psychosocial working environment was associated with sickness absence of all lengths. Looking at individual factors, reducing exposure to bullying and perceived stress and increasing skill discretion seemed most promising for reducing sickness absence. Improving the psychosocial working environment may hold great potential for reducing sickness absence rates in public hospitals.

## Supplementary data

Supplementary data are available at *EURPUB* online.

## Funding

The work was supported by the Danish Regions (Danske Regioner; employer organization) and The Danish Association of Local Government Employees Organizations (Forhandlingsfællesskabet; employee organization). N.H.R. received funding from the Working Environment Foundation (grant no. 13-2015-09). The sponsors had no role in the study design, the collection, analysis and interpretation of the data, the writing of the manuscript or the decision to submit it for publication.

*Conflicts of interest*: None declared.

Key points

- Many previous studies have examined the effect of one or a few psychosocial work factors on sickness absence, but the preventive potential associated with improvement in several factors simultaneously has rarely been estimated.
- Using observational data and simulation-based methods, we found that general improvements in the psychosocial working environment were associated with lower sickness absence rates in this cohort of 24 990 public hospital employees.
- Reducing exposure to bullying and perceived stress and increasing skill discretion were the most important factors for reducing sickness absence.
- The preventive potential was, in general, similar for short- intermediate- and long-term sickness absence rates.
- Our results provide decision-makers with guidance on identifying potential targets for work environment interventions aiming at reducing sickness absence rates.

## Supplementary Material

ckac109_Supplementary_Data

## Data Availability

Anonymized data from the WHALE cohort are available upon reasonable request through collaborative agreements. Please contact Professor Naja Hulvej Rod (nahuro@sund.ku.dk). Data used in this study from Danish registers are not publicly available.
