# Supplementary material for: Impact of hypothetical improvements in the psychosocial work environment on sickness absence rates: a simulation study
Source: Eur J Public Health. 2022 Aug 27;32(5):716–22. doi: 10.1093/eurpub/ckac109 (PMC9527953; doi:10.1093/eurpub/ckac109)
Supplement: ckac109_Supplementary_Data [file ckac109_supplementary_data.docx]

**Supplemental Material**

**Title**: Impact of hypothetical improvements in the psychosocial work environment on sickness absence rates: a simulation study

**Authors:** Jimmi Mathisen^1,3^, Tri-Long Nguyen^1^, Johan Høy Jensen^2,3^, Amar Jayant Mehta^1,3^, Reiner Rugulies^1,3,4,5^, Naja Hulvej Rod^1^

**Affiliations:**

1: Section of Epidemiology, Department of Public Health, University of Copenhagen, Copenhagen, Denmark
2: Department of Occupational and Environmental Medicine, Bispebjerg and Frederiksberg Hospital, University of Copenhagen, Copenhagen, Denmark
3: Copenhagen Stress Research Center, Copenhagen, Denmark
4: National Research Centre for the Working Environment, Copenhagen, Denmark
5: Department of Psychology, University of Copenhagen, Copenhagen, Denmark

**Corresponding author:**Name: Jimmi Mathisen
Institution: Department of Public Health, University of Copenhagen

Address: Oester Farimagsgade 5, P.O. Box 2099, 1014 Copenhagen, Denmark

Telephone number: +45 35 33 53 39

Email: [jima@sund.ku.dk](mailto:jima@sund.ku.dk)

ORCID: 0000-0002-7391-7296

**Table of contents:**

S1: Characteristics of the work environment scales and included items.

S2: Elaboration of causal issues with splitting up sickness absence by length and correction via inverse probability weighting

S3: Supplemental tables

| **Psychosocial working conditions** | | | | | |
| --- | --- | --- | --- | --- | --- |
| **Scale** | **Cronbach's alpha** | **n missing^a^** | **Phrasing of item** *To what extent…* | **COPSOQ-II scale^b^ (n items in scale)** | **Corresponding COPSOQ-II item** |
| Collaboration | 0.72 | 136 | … are you and your colleagues good at coming up with suggestions for improving work procedures? | Social community at work (3) | 33.5 |
|  |  |  | … do you and your colleagues take responsibility for a nice atmosphere and tone of communication? |  | 33.4 |
|  |  |  | … do you get help and support from your colleagues when needed?^c^ |  | 33.1 |
| Influence on schedule |  | 456 | … are you able to schedule your work time, so that you can take into account private matters | - | 32.18 |
| Influence on work |  | 87 | … do you have influence on how you do your work? | Influence (4) | 32.4 / 32.13 |
| Inclusiveness |  | 400 | … do you and your colleagues give space for each other’s differences at your workplace? (e.g. regarding sex, age and background) | Inclusiveness | 36.3, 36.13 |
| Justice | 0.81 | 1481 | … are conflicts resolved in a fair way? | Justice and respect (4) | 36.5 |
|  |  |  | … is the work distributed fairly? |  | 36.25 |
| Leadership quality | 0.91 | 1300 | … would you say that your immediate supervisor is good at organizing work | Quality of leadership (4) | 48.2 |
|  |  |  | … would you say that your immediate supervisor gives high priority to workplace wellbeing |  | 48.4 |
|  |  |  | … do you get help and support by your nearest supervisor when needed? |  | 47.2 |
|  |  |  | … is your work recognized and appreciated by the management? |  | 35.10 |
| Predictability |  | 581 | … are you informed well in advance concerning for example important decisions, changes, or plans for the future? | Predictability (2) | 35.6 |
| Role clarity |  | 578 | … does your work have clear objectives? | Role clarity (3) | 35.7 |
| Skill discretion |  | 83 | … do you have the possibility of learning new things through your work? | Possibilities for development (4) | 35.31 |
| Trust | 0.85 | 462 | … can you trust the information that comes from the management? | Vertical trust (4) | 36.4 |
|  |  |  | … does the management trust the employees to do their work well? |  | 36.1 |
| Work demands | 0.72 | 0 | … do you have time for breaks throughout your workday?^d^ | Quantitative demands (4) | - |
|  |  |  | … do you have enough time for your work tasks? |  | 32.23 |
| Bullying |  | 415 | Have you been exposed to bullying during the past 12 months? |  |  |
| Sexual harassment |  | 456 | Have you been exposed to unwanted sexual attention at your workplace during the past 12 months? |  |  |
| Threats |  | 257 | Have you been exposed to threats of violence at your workplace during the past 12 months? |  |  |
| Violence |  | 232 | Have you been exposed to physical violence at your workplace during the past 12 months? |  |  |
| **Cognitive and emotional reactions** | | | | | |
| **Scale** | **Cronbach's alpha** | **% missing** | *To what extent…* | **COPSOQ-II scale (n items in scale)** | **COPSOQ-II item** |
| Job satisfaction | 0.90 | 165 | … are you pleased with your job as a whole, everything taken into consideration? | Job satisfaction (4) | 34.6 |
|  |  |  | … are you pleased with your work prospects? |  | 34.1 |
|  |  |  | … are you pleased with the way your abilities are used? |  | 34.4 |
|  |  |  | … are you pleased with the work environment? |  | 34.2 |
| Perceived stress^e^ |  | 497 | … have you been stressed during the past six months? |  | 10.30 |

**Supplemental Material 1: Characteristics of the work environment scales and included items.**

1. Employees were defined as having a missing value on a scale if they were missing on half or more of the included items.
2. Pejtersen JH, Kristensen TS, Borg V, Bjorner JB. The second version of the Copenhagen Psychosocial Questionnaire. Scand J Public Health. 2010 Feb;38(3_suppl):8–24.
3. Used in the COPSOQ-II scale *social support from colleagues*.
4. Not part of the *quantitative demands* scale in COPSOQ-II
5. Responses categorized as *Low* (no), *Medium* (Yes, sometimes + Yes, monthly), *High* (Yes, weekly + Yes, daily).

**Supplemental Material 2: Elaboration of causal issues with splitting up sickness absence by length and correction via inverse probability weighting**

In this study, we separately analysed three types of sickness absence defined by their length. As we divide the sickness absence into periods of distinct lengths, we risk biasing the association between the work environment covariates and the sickness absence type in question. Here, we present the issue using language and illustrations from the theory of Directed Acyclic Graphs (DAG) (1). Briefly, DAGs are a graphical tool for identifying and depicting assumptions about causal associations (including biasing associations) between variables. We use the tool DAGgity to illustrate the DAGs (2). Green nodes denote exposure covariate sets, white nodes denote statistically adjusted covariate sets and blue nodes denote outcome variables. Arrows denote assumed causal associations. We also present sensitivity analyses to correct for the issue which makes use of Inverse Probability Weights (3).

In the main analysis of short-, intermediate- and long-term sickness absence, we implicitly assume that the three types are independent of each other. This is illustrated in the graph below (Figure S1), which shows the work environment factors (working conditions and cognitive and emotional reactions) as the exposure covariate set, sociodemographic, workplace, and employment characteristics as adjusted potential confounders and short-, intermediate, and long-term sickness absence as three distinct outcomes with no casual association between them. Under these assumptions, it is meaningful to analyse the three sickness absence types separately (as we did in the main analysis).

**Figure S1: Independence between short-, intermediate-, and long-term sickness absence.**


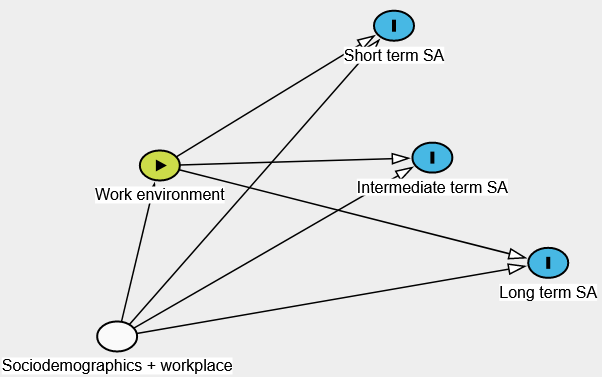


It is a strong assumption, however, that the three types of sickness absence are independent of each other. In our analysis, associations between them can arise from at least two processes: First, short-term sickness absence is associated with future longer sickness absence periods independently of underlying disease (Hultin et al. 2012). Second, the three outcomes are associated because they are mutually exclusive. That is, if a sickness absence period is defined as short-term, it cannot be intermediate-, or long-term. The associations under the assumptions of dependence are illustrated in Figure S2.

**Figure S2: Dependence between short-, intermediate-, and long-term sickness absence.**


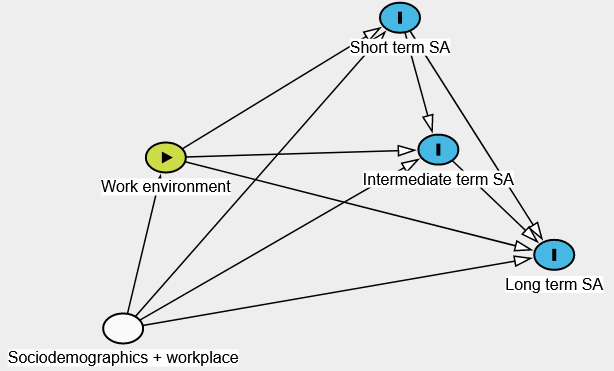


In this graph, the three types of sickness absence are assumed to be causally associated with each other: short-term sickness absence influences intermediate-, and long-term sickness absence, and intermediate-term sickness absence also influence long-term sickness absence. If this causal structure holds then estimating associations between work environment covariates and each of these outcomes separately results in effectively conditioning on mediators (that is, variables on the causal pathway) or colliders (that is, common effects of exposure and outcome), leading to biased covariate-outcome estimates. For example, the analysis of the association between the work environment and intermediate-term sickness absence requires conditioning on short-term sickness absence (mediator) and long-term sickness absence (collider). Conditioning on mediators implies adjusting for events on the causal pathway taking a specific value. This “removes” some of the association between the exposure and the outcome. Conditioning on colliders implies adjusting for future events related to both the exposure and outcome taking a specific value. This creates spurious associations between the exposure and the outcome through collider-stratification bias.

To correct for this potential bias, we applied inverse probability weights to the analysis of each sickness absence type. Inverse probability weights are used to create a pseudo-population in which the covariate-outcome association is independent of biasing covariates (49). This corresponds to removing the associations which give rise to bias in the data at hand. Briefly, the procedure was as follows: First, for each sickness absence type, we fitted a propensity score model by logistic regression to estimate the probability of not having the two complementary sickness absence types. For example, for the analyses of short-term sickness absence, we estimated the probability of not having had intermediate- or long-term sickness absence. The probabilities were estimated given sociodemographic, workplace and employment characteristics, working conditions, and cognitive and emotional reactions. For the weights used in the analyses of short-, and intermediate-term sickness absence, the rate of these absence types, respectively, were also used as predictors. Second, we constructed the inverse probability weights as the reverse of the constructed propensity scores using the R package *ipw*. To address extreme weights, we truncated all weights at the 0.5^th^ and 99.5^th^ percentile (Cole and Hernan 2008). Finally, we applied the weights to the regression models for each outcome and estimated contrasts similar to those in the main analyses. Below we describe how each set of weights were created.

Short-term absence

Analysing short-term sickness absence periods excludes intermediate- and long-term periods. That is, a sickness absence period can only be short-term if it did not develop into intermediate-, or long-term sickness absence. By analysing the associations between work environment covariates and short-term sickness absence, we thereby condition on future events (intermediate- or long-term sickness absence) not taking place. This is illustrated in the graph below (Figure S3)

**Figure S3: Naïve analysis of the association between the work environment and short-term sickness absence.**


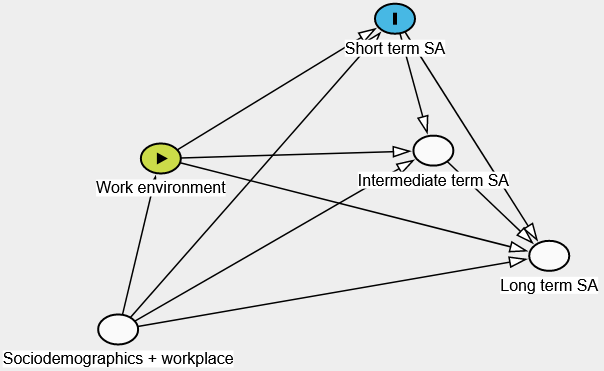


To correct these biasing associations, we applied weights based on the inverse of the probability that each employee had no intermediate-, or long-term sickness absence. This probability was estimated using sociodemographic, workplace and employment characteristics, working conditions, cognitive and emotional reactions as well as the short-term sickness absence rate. Applying these weights in the analysis between the work environment and short-term sickness absence results in the causal structure illustrated below in Figure S4.

**Figure S4: Inverse probability weighted analysis of the association between the work environment and short-term sickness absence.**


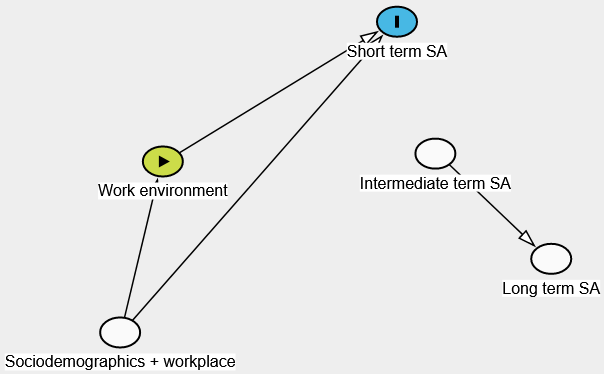


As shown in Figure S4, estimating the association between the work environment and short-term sickness absence using IP weights does not imply adjusting for colliders, as intermediate-, and long-term sickness absence are no longer associated with either the work environment or short-term sickness absence, and therefore are no longer colliders.

**Figure S5: Density plot (log scale) of weights used in the analysis of short-term sickness absence.**


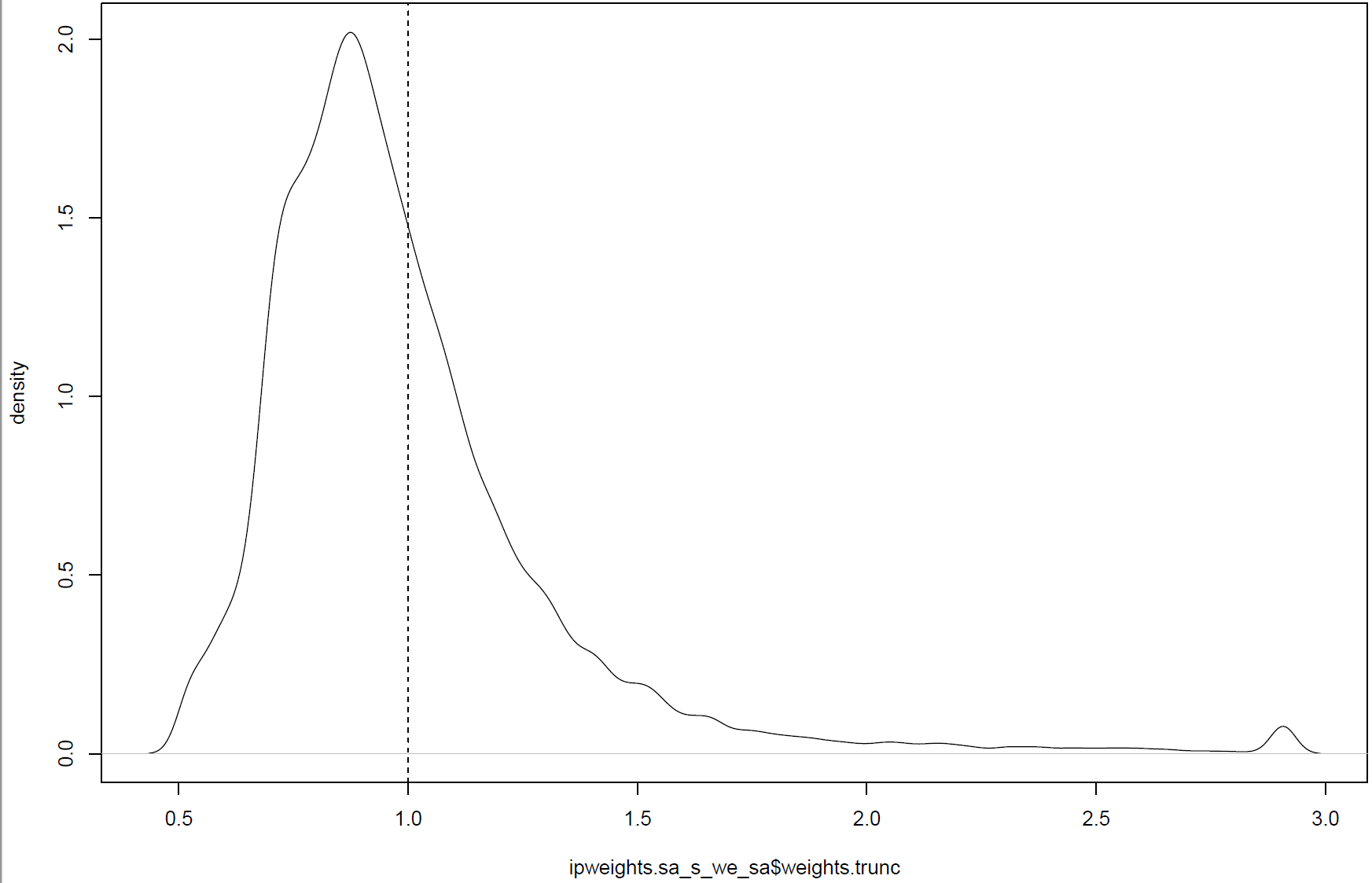


Intermediate-term absence

Analysing intermediate-term sickness absence periods excludes short- and long-term periods. That is, a sickness period can only be intermediate-term if it exceeded a short-term period but did not develop into a long-term period. By analysing the association between the work environment and intermediate-term sickness absence, we thereby condition on prior (short-term) and future (long-term) events not taking place. This is illustrated in the graph below (Figure S6).

**Figure S6: Naïve analysis of the association between the work environment and intermediate-term sickness absence.**


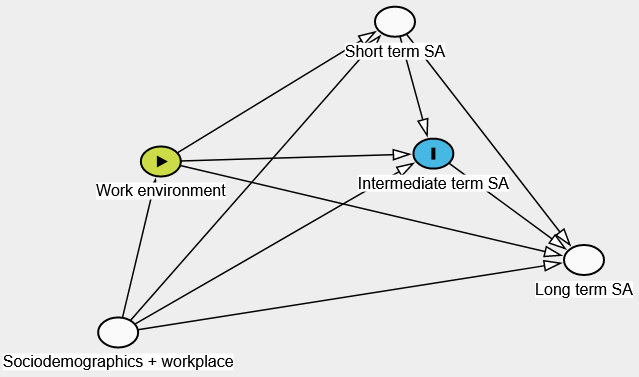


To correct these biasing associations, we applied weights based on the inverse of the probability that each employee had no short-, or long-term sickness absence. This probability was estimated using sociodemographic, workplace and employment characteristics, working conditions, cognitive and emotional reactions, as well as the intermediate-term sickness absence rate. Applying these weights in the analysis between the work environment and intermediate-term sickness absence results in the causal structure illustrated below in Figure S7.

**Figure S7: Inverse probability weighted analysis of the association between the work environment and intermediate-term sickness absence.**


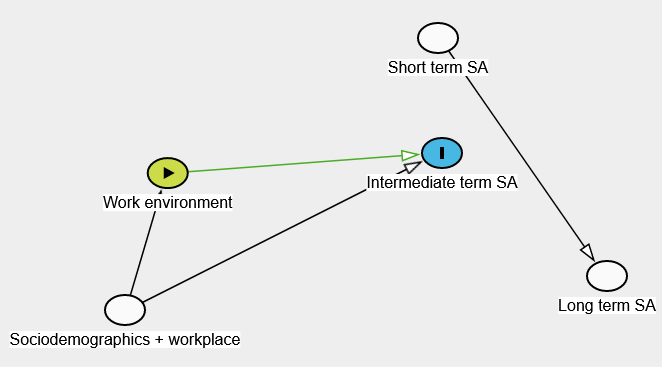


As shown in Figure S7, estimating the association between the work environment and intermediate-term sickness absence using IP weights does not imply adjustment for mediators or colliders.

**Figure S8: Density plot (log scale) of weights used in the analysis of intermediate-term sickness absence.**


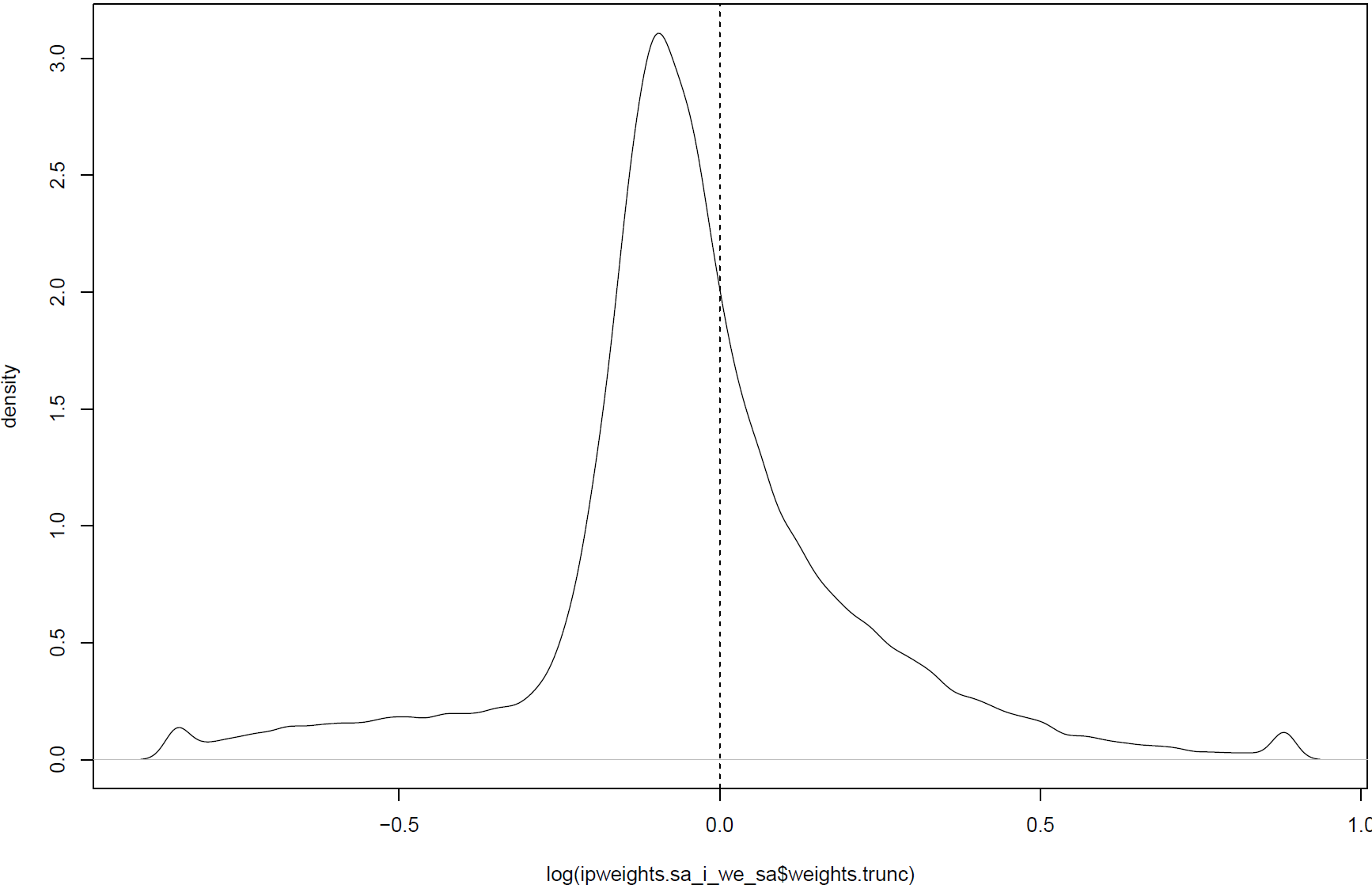


Long-term sickness absence

If one defines a sickness absence period as long-term, it results in excluding short- or intermediate-term sickness absence. That is, a sickness absence period can only be long-term if it exceeded both short-, and intermediate-term periods. By analysing the association between the work environment and long-term sickness absence, we thereby condition on prior non-events (Figure S9).

**Figure S9: Naïve analysis of the association between the work environment and long-term sickness absence.**


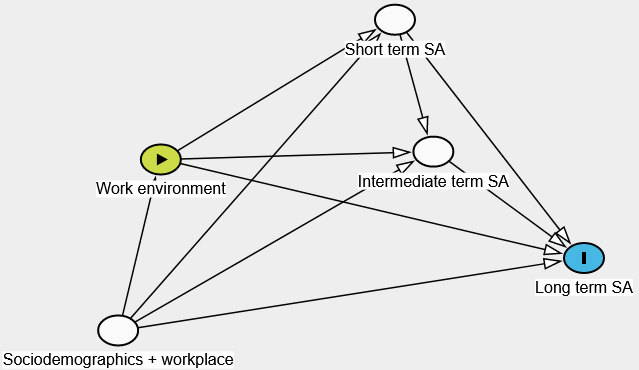


To correct for this, we applied weights based on the inverse of the probability that each employee had no short-, or intermediate-term sickness absence. This probability was estimated using sociodemographic, workplace and employment characteristics, working conditions as well as cognitive and emotional reactions. Applying these weights in the analysis between the work environment and long-term sickness absence results in the causal structure illustrated below in Figure S10.

**Figure S10: Inverse probability weighted analysis of the association between the work environment and long-term sickness absence.**


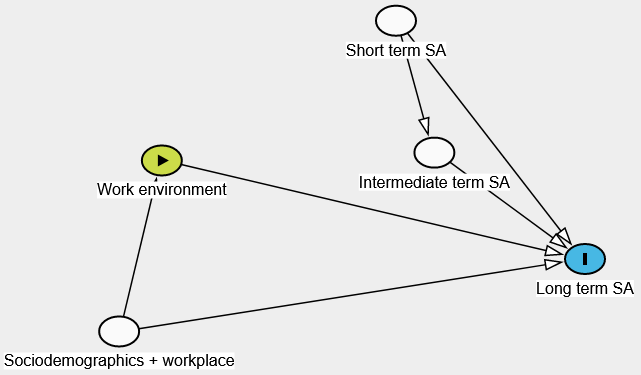


As shown in Figure S10, estimating the association between the work environment and long-term sickness absence using IP weights implies no mediator adjustment, as short-, and intermediate-term sickness absence are no longer on the causal pathway between the work environment and long-term sickness absence.

**Figure S11: Density plot (logscale) of weights used in the analysis of intermediate-term sickness absence.**
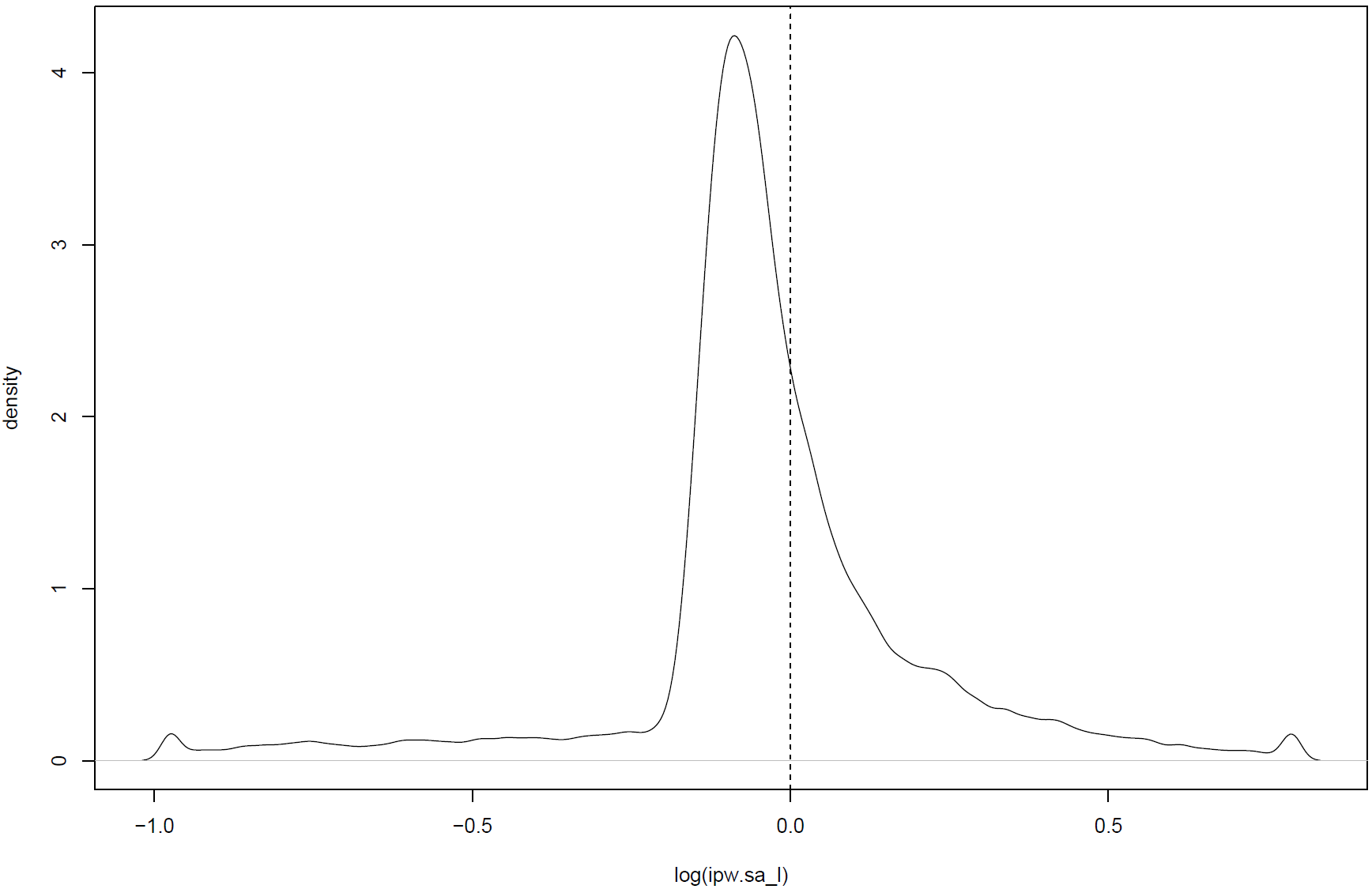


Results

The results are shown in Supplemental Material 3, Table S8. Overall, the results were similar to those of the main analysis shown in Figure 3 and Table S2. Contrasting the scenarios where all employees were set to the most desirable work environment compared with the least desirable work environment showed slightly lower rate ratios of short-term sickness absence (RR: 0.51, 95% CI: 0.50 – 0.52) and slightly higher intermediate-term sickness absence (RR: 0.44, 95% CI: 0.43 – 0.45) than those in the main analysis.

REFERENCES

1. Greenland S, Pearl J, Robins JM. Causal Diagrams for Epidemiologic Research. Epidemiology. 1999;10(1):37–48.

2. Textor J, Hardt J, Knüppel S. DAGitty: A Graphical Tool for Analyzing Causal Diagrams. Epidemiology. 2011 Sep;22(5):745.

3. Cole SR, Hernan MA. Constructing Inverse Probability Weights for Marginal Structural Models. American Journal of Epidemiology. 2008 Jul 15;168(6):656–64.

**Supplemental Material 3: Supplemental tables**

**Table S1:** Descriptive psychosocial work characteristics of 24 990 employees and their mean total, short-, intermediate-, and long-term sickness absence rates.

|  | Level | N | % of N | % Total sickness  absence | % short-term sickness  absence | % intermediate-term sickness  absence | % long-term sickness  absence |
| --- | --- | --- | --- | --- | --- | --- | --- |
| **Total** | - | 24990 | 100 | 4.0 | 1.5 | 1.3 | 1.2 |
| **Psychosocial work environment** | | | | | | | |
| Collaboration | Low | 6405 | 26 | 4.9 | 1.6 | 1.5 | 1.8 |
|  | Medium | 11899 | 48 | 3.7 | 1.5 | 1.2 | 1.0 |
|  | High | 6686 | 27 | 3.7 | 1.5 | 1.2 | 1.0 |
| Influence on schedule | Low | 3701 | 15 | 5.0 | 1.7 | 1.6 | 1.8 |
|  | Medium | 17203 | 69 | 4.0 | 1.5 | 1.3 | 1.2 |
|  | High | 4086 | 16 | 3.2 | 1.3 | 1.1 | 0.9 |
| Influence on work | Low | 9349 | 37 | 4.9 | 1.7 | 1.5 | 1.7 |
|  | Medium | 10291 | 41 | 3.7 | 1.5 | 1.2 | 1.0 |
|  | High | 5350 | 21 | 3.0 | 1.2 | 1.0 | 0.8 |
| Inclusiveness | Low | 6577 | 26 | 4.8 | 1.6 | 1.5 | 1.7 |
|  | Medium | 13135 | 53 | 3.8 | 1.5 | 1.2 | 1.1 |
|  | High | 5278 | 21 | 3.5 | 1.4 | 1.2 | 0.9 |
| Justice | Low | 6122 | 25 | 5.1 | 1.7 | 1.5 | 1.9 |
|  | Medium | 12174 | 49 | 3.9 | 1.5 | 1.2 | 1.1 |
|  | High | 6694 | 27 | 3.2 | 1.3 | 1.1 | 0.8 |
| Leadership quality | Low | 5724 | 23 | 5.2 | 1.7 | 1.6 | 2.0 |
|  | Medium | 13700 | 55 | 3.7 | 1.5 | 1.2 | 1.0 |
|  | High | 5566 | 22 | 3.5 | 1.4 | 1.1 | 1.0 |
| Predictability | Low | 5990 | 24 | 5.1 | 1.7 | 1.5 | 1.9 |
|  | Medium | 11411 | 46 | 3.9 | 1.5 | 1.3 | 1.1 |
|  | High | 7589 | 30 | 3.3 | 1.4 | 1.1 | 0.9 |
| Role clarity | Low | 2902 | 12 | 5.1 | 1.7 | 1.5 | 1.9 |
|  | Medium | 19680 | 79 | 3.9 | 1.5 | 1.3 | 1.2 |
|  | High | 2408 | 10 | 3.1 | 1.3 | 1.0 | 0.8 |
| Skill discretion | Low | 9647 | 39 | 4.8 | 1.7 | 1.5 | 1.6 |
|  | Medium | 9866 | 39 | 3.8 | 1.5 | 1.2 | 1.1 |
|  | High | 5477 | 22 | 2.9 | 1.2 | 1.0 | 0.7 |
| Trust | Low | 5578 | 22 | 5.2 | 1.7 | 1.6 | 2.0 |
|  | Medium | 14435 | 58 | 3.8 | 1.5 | 1.2 | 1.1 |
|  | High | 4977 | 20 | 3.3 | 1.3 | 1.1 | 0.9 |
| Work demands | Low | 9197 | 37 | 4.5 | 1.6 | 1.4 | 1.6 |
|  | Medium | 8455 | 34 | 3.7 | 1.5 | 1.2 | 1.1 |
|  | High | 7338 | 29 | 3.6 | 1.5 | 1.2 | 1.0 |
| Bullying. last 12 months | Yes | 2594 | 10 | 6.1 | 1.8 | 1.9 | 2.4 |
|  | No | 22396 | 90 | 3.8 | 1.5 | 1.2 | 1.1 |
| Sexual harassment. last 12 months | Yes | 1135 | 5 | 5.4 | 1.9 | 1.9 | 1.6 |
|  | No | 23855 | 95 | 3.9 | 1.5 | 1.2 | 1.2 |
| Threats. last 12 months | Yes | 3227 | 13 | 5.3 | 1.8 | 1.9 | 1.7 |
|  | No | 21763 | 87 | 3.8 | 1.5 | 1.2 | 1.2 |
| Violence. last 12 months | Yes | 1998 | 8 | 5.2 | 1.8 | 1.8 | 1.5 |
|  | No | 22992 | 92 | 3.9 | 1.5 | 1.2 | 1.2 |
| **Cognitive and emotional reactions** | | | | | | | |
| Job satisfaction | Low | 6097 | 24 | 5.6 | 1.8 | 1.6 | 2.2 |
|  | Medium | 11041 | 44 | 3.8 | 1.5 | 1.2 | 1.0 |
|  | High | 7852 | 31 | 3.0 | 1.2 | 1.0 | 0.8 |
| Perceived stress. last 6 months^a^ | Low | 6824 | 27 | 3.1 | 1.3 | 1.0 | 0.8 |
|  | Medium | 11948 | 48 | 3.7 | 1.5 | 1.2 | 1.0 |
|  | High | 6218 | 25 | 5.6 | 1.7 | 1.7 | 2.2 |

**Table S2:** Rate ratios and 95% confidence intervals of sickness absence of different lengths in **etiologic** contrast scenarios (most desirable vs least desirable levels of psychosocial work factors). N=24 990

|  | Total sickness  absence | | | Short-term  sickness  absence | | | Intermediate-term sickness  absence | | | Long-term  sickness  absence | | |  |
| --- | --- | --- | --- | --- | --- | --- | --- | --- | --- | --- | --- | --- | --- |
|  | RR | 95% CI,  lower | 95% CI,  higher | RR | 95% CI,  lower | 95% CI,  higher | RR | 95% CI,  lower | 95% CI,  higher | RR | 95% CI,  lower | 95%CI,  higher | |
| All improvements | **0.46** | 0.40 | 0.52 | **0.59** | 0.54 | 0.63 | **0.54** | 0.48 | 0.60 | **0.47** | 0.34 | 0.61 | |
| **Psychosocial working conditions** | | | | | | | | | | | | | |
| Bullying, last 12 months | **0.86** | 0.83 | 0.90 | **0.92** | 0.89 | 0.94 | **0.87** | 0.83 | 0.90 | **0.85** | 0.78 | 0.92 | |
| Collaboration | **1.00** | 0.98 | 1.02 | **1.02** | 1.00 | 1.03 | **0.99** | 0.98 | 1.01 | **0.99** | 0.94 | 1.03 | |
| Inclusiveness | **0.96** | 0.95 | 0.98 | **0.99** | 0.97 | 1.00 | **0.99** | 0.97 | 1.01 | **0.95** | 0.90 | 0.99 | |
| Influence on schedule | **0.96** | 0.94 | 0.99 | **0.96** | 0.94 | 0.97 | **0.97** | 0.95 | 1.00 | **0.96** | 0.90 | 1.01 | |
| Influence on work | **0.93** | 0.91 | 0.96 | **0.91** | 0.89 | 0.92 | **0.96** | 0.94 | 0.98 | **0.95** | 0.90 | 1.00 | |
| Justice | **0.94** | 0.92 | 0.96 | **0.95** | 0.93 | 0.96 | **0.96** | 0.95 | 0.98 | **0.93** | 0.90 | 0.97 | |
| Leadership quality | **0.98** | 0.96 | 1.00 | **0.99** | 0.98 | 1.01 | **0.98** | 0.97 | 1.00 | **0.98** | 0.94 | 1.02 | |
| Predictability | **0.95** | 0.94 | 0.97 | **0.97** | 0.95 | 0.98 | **0.96** | 0.94 | 0.98 | **0.96** | 0.92 | 0.99 | |
| Role clarity | **0.96** | 0.93 | 0.99 | **0.98** | 0.95 | 1.00 | **0.96** | 0.93 | 0.99 | **0.96** | 0.90 | 1.03 | |
| Sexual harassment, last 12 months | **0.94** | 0.90 | 0.98 | **0.95** | 0.92 | 0.98 | **0.92** | 0.88 | 0.97 | **0.98** | 0.89 | 1.07 | |
| Skill discretion | **0.91** | 0.89 | 0.94 | **0.92** | 0.91 | 0.94 | **0.93** | 0.91 | 0.96 | **0.91** | 0.86 | 0.95 | |
| Threats, last 12 months | **0.94** | 0.91 | 0.97 | **0.97** | 0.95 | 0.99 | **0.91** | 0.89 | 0.94 | **0.97** | 0.91 | 1.02 | |
| Trust | **0.96** | 0.94 | 0.99 | **0.97** | 0.95 | 0.98 | **0.99** | 0.97 | 1.01 | **0.95** | 0.90 | 1.00 | |
| Violence, last 12 months | **0.96** | 0.93 | 0.99 | **0.95** | 0.92 | 0.97 | **0.92** | 0.89 | 0.95 | **1.04** | 0.97 | 1.12 | |
| Work demands | **0.97** | 0.95 | 0.99 | **1.02** | 1.00 | 1.03 | **0.99** | 0.97 | 1.01 | **0.95** | 0.91 | 0.99 | |
| **Cognitive and emotional reactions** |  |  |  |  |  |  |  |  |  |  |  |  | |
| Job satisfaction | **0.93** | 0.91 | 0.95 | **0.95** | 0.94 | 0.96 | **0.95** | 0.93 | 0.97 | **0.92** | 0.88 | 0.96 | |
| Perceived stress. last 6 months | **0.90** | 0.87 | 0.92 | **0.92** | 0.90 | 0.93 | **0.92** | 0.89 | 0.94 | **0.91** | 0.86 | 0.96 | |

Estimates of *All improvements* and *psychosocial working conditions* are adjusted for: Age, sex, occupational group, household income, marital status, part-time/full-time employment, seniority and workplace. Estimates of *all cognitive and emotional reactions* are adjusted for the above mentioned factors and also *psychosocial working conditions.*

**Table S3:** Rate ratios and 95% confidence intervals of sickness absence of different lengths in **realistic** contrast scenarios (most desirable vs observed levels of psychosocial work factors). N=24 990

|  | Total sickness  absence | | | Short-term  sickness  absence | | | Intermediate-term sickness  absence | | | Long-term  sickness  absence | | |  |
| --- | --- | --- | --- | --- | --- | --- | --- | --- | --- | --- | --- | --- | --- |
|  | RR | 95% CI,  lower | 95% CI,  higher | RR | 95% CI,  lower | 95% CI,  higher | RR | 95% CI,  lower | 95% CI,  higher | RR | 95% CI,  lower | 95% CI,  higher | |
| All improvements | **0.70** | 0.64 | 0.76 | **0.74** | 0.70 | 0.78 | **0.78** | 0.71 | 0.85 | **0.65** | 0.52 | 0.79 | |
| **Psychosocial working conditions** | | | | | | | | | | | | | |
| Bullying, last 12 months | **0.98** | 0.98 | 0.99 | **0.99** | 0.99 | 0.99 | **0.98** | 0.98 | 0.99 | **0.97** | 0.96 | 0.99 | |
| Collaboration | **1.02** | 1.00 | 1.03 | **1.02** | 1.01 | 1.03 | **1.01** | 0.99 | 1.02 | **1.01** | 0.97 | 1.05 | |
| Inclusiveness | **0.98** | 0.97 | 1.00 | **1.00** | 0.98 | 1.01 | **1.01** | 0.98 | 1.03 | **0.96** | 0.92 | 1.00 | |
| Influence on schedule | **0.99** | 0.96 | 1.01 | **0.97** | 0.96 | 0.99 | **0.99** | 0.97 | 1.02 | **0.98** | 0.92 | 1.03 | |
| Influence on work | **0.96** | 0.94 | 0.98 | **0.94** | 0.92 | 0.95 | **0.98** | 0.96 | 1.00 | **0.98** | 0.93 | 1.02 | |
| Justice | **0.97** | 0.95 | 0.99 | **0.97** | 0.96 | 0.98 | **0.98** | 0.97 | 1.00 | **0.96** | 0.93 | 0.99 | |
| Leadership quality | **1.00** | 0.99 | 1.02 | **1.00** | 0.99 | 1.02 | **1.00** | 0.99 | 1.02 | **1.02** | 0.97 | 1.06 | |
| Predictability | **0.98** | 0.97 | 1.00 | **0.99** | 0.98 | 1.00 | **0.98** | 0.97 | 1.00 | **0.98** | 0.94 | 1.01 | |
| Role clarity | **0.97** | 0.94 | 1.00 | **0.99** | 0.96 | 1.01 | **0.97** | 0.93 | 1.00 | **0.98** | 0.91 | 1.05 | |
| Sexual harassment, last 12 months | **1.00** | 0.99 | 1.00 | **1.00** | 0.99 | 1.00 | **0.99** | 0.99 | 1.00 | **1.00** | 0.99 | 1.00 | |
| Skill discretion | **0.94** | 0.92 | 0.96 | **0.95** | 0.93 | 0.96 | **0.96** | 0.94 | 0.98 | **0.93** | 0.88 | 0.97 | |
| Threats, last 12 months | **0.99** | 0.98 | 0.99 | **0.99** | 0.99 | 1.00 | **0.98** | 0.98 | 0.99 | **0.99** | 0.98 | 1.00 | |
| Trust | **1.00** | 0.98 | 1.02 | **0.99** | 0.97 | 1.00 | **1.01** | 0.99 | 1.03 | **0.99** | 0.94 | 1.04 | |
| Violence, last 12 months | **0.99** | 0.99 | 1.00 | **0.99** | 0.99 | 1.00 | **0.99** | 0.99 | 0.99 | **1.00** | 0.99 | 1.01 | |
| Work demands | **0.99** | 0.98 | 1.00 | **1.02** | 1.00 | 1.03 | **1.00** | 0.99 | 1.02 | **0.97** | 0.94 | 1.01 | |
| **Cognitive and emotional reactions** |  |  |  |  |  |  |  |  |  |  |  |  | |
| Job satisfaction | **0.97** | 0.95 | 0.98 | **0.97** | 0.96 | 0.98 | **0.97** | 0.96 | 0.99 | **0.96** | 0.93 | 1.00 | |
| Perceived stress, last 6 months | **0.95** | 0.93 | 0.97 | **0.95** | 0.94 | 0.96 | **0.95** | 0.93 | 0.97 | **0.98** | 0.94 | 1.01 | |

Estimates of *All improvements* and *psychosocial working conditions* are adjusted for: Age, sex, occupational group, household income, marital status, part-time/full-time employment, seniority and workplace. Estimates of *all cognitive and emotional reactions* are adjusted for the above mentioned factors and also *psychosocial working conditions.*

**Table S4:** Percentual changes (95% confidence intervals) in sickness absence rates from realistic improvements. N=24 990

|  | Total sickness  absence | | | Short-term  sickness  absence | | | Intermediate-term sickness  absence | | | Long-term  sickness  absence | | |  |
| --- | --- | --- | --- | --- | --- | --- | --- | --- | --- | --- | --- | --- | --- |
|  | % change | 95% CI,  lower | 95% CI,  higher | % change | 95% CI,  lower | 95% CI,  higher | % change | 95% CI,  lower | 95% CI,  higher | % change | 95% CI,  lower | 95% CI,  higher | |
| All improvements | **-30** | 36 | -24 | **-26** | -30 | -22 | **-22** | -29 | -15 | **-35** | -48 | -21 | |
| **Psychosocial working conditions** | | | | | | | | | | | | | |
| Bullying. last 12 months | **-2** | -2 | -1 | **-1** | -1 | -1 | **-2** | -2 | -1 | **-3** | -4 | -1 | |
| Collaboration | **+2** | 0 | +3 | **+2** | +1 | +3 | **+1** | -1 | +2 | **+1** | -3 | +5 | |
| Inclusiveness | **-2** | -3 | 0 | **0** | -2 | +1 | **+1** | -2 | +3 | **-4** | -8 | 0 | |
| Influence on schedule | **-1** | -4 | +1 | **-3** | -4 | -1 | **-1** | -3 | +2 | **-2** | -8 | +3 | |
| Influence on work | **-4** | -6 | -2 | **-6** | -8 | -5 | **-2** | -4 | 0 | **-2** | -7 | 2 | |
| Justice | **-3** | -5 | -1 | **-3** | -4 | -2 | **-2** | -3 | 0 | **-4** | -7 | -1 | |
| Leadership quality | **0** | -1 | +2 | **0** | -1 | +2 | **0** | -1 | +2 | **2** | -3 | +6 | |
| Predictability | **-2** | -3 | 0 | **-1** | -2 | 0 | **-2** | -3 | 0 | **-2** | -6 | +1 | |
| Role clarity | **-3** | -6 | 0 | **-1** | -4 | +1 | **-3** | -7 | 0 | **-2** | -9 | +5 | |
| Sexual harassment. last 12 months | **0** | -1 | 0 | **0** | -1 | 0 | **-1** | -1 | 0 | **0** | -1 | 0 | |
| Skill discretion | **-6** | -8 | -4 | **-5** | -7 | -4 | **-4** | -6 | -2 | **-7** | -12 | -3 | |
| Threats. last 12 months | **-1** | -2 | -1 | **-1** | -1 | 0 | **-2** | -2 | -1 | **-1** | -2 | 0 | |
| Trust | **0** | -2 | +2 | **-1** | -3 | 0 | **1** | -1 | +3 | **-1** | -6 | +4 | |
| Violence. last 12 months | **-1** | -1 | 0 | **-1** | -1 | 0 | **-1** | -1 | -1 | **0** | -1 | +1 | |
| Work demands | **-1** | -2 | 0 | **2** | 0 | +3 | **0** | -1 | +2 | **-3** | -6 | +1 | |
| **Cognitive and emotional reactions** |  |  |  |  |  |  |  |  |  |  |  |  | |
| Job satisfaction | **-3** | -5 | -2 | **-3** | -4 | -2 | **-3** | -4 | -1 | **-4** | -7 | 0 | |
| Perceived stress. last 6 months | **-5** | -7 | -3 | **-5** | -6 | -4 | **-5** | -7 | -3 | **-2** | -6 | +1 | |

Estimates of *All improvements* and *psychosocial working conditions* are adjusted for: Age, sex, occupational group, household income, marital status, part-time/full-time employment, seniority and workplace. Estimates of *all cognitive and emotional reactions* are adjusted for the above mentioned factors and also *psychosocial working conditions.*

**Table S5:** Rate ratios and 95% confidence intervals of sickness absence of different lengths in etiologic contrast scenarios for **women**. N=19 674

|  | Total sickness  absence | | | Short-term  sickness  absence | | | Intermediate-term sickness  absence | | | Long-term  sickness  absence | | |  |
| --- | --- | --- | --- | --- | --- | --- | --- | --- | --- | --- | --- | --- | --- |
|  | RR | 95% CI,  lower | 95% CI,  higher | RR | 95% CI,  lower | 95% CI,  higher | RR | 95% CI,  lower | 95% CI,  higher | RR | 95% CI,  lower | 95% CI,  higher | |
| All improvements | **0.46** | 0.40 | 0.52 | **0.59** | 0.55 | 0.63 | **0.54** | 0.48 | 0.60 | **0.47** | 0.33 | 0.61 | |
| **Psychosocial working conditions** | | | | | | | | | | | | | |
| Bullying, last 12 months | **0.86** | 0.82 | 0.89 | **0.91** | 0.89 | 0.93 | **0.86** | 0.83 | 0.90 | **0.85** | 0.77 | 0.92 | |
| Collaboration | **1.00** | 0.98 | 1.02 | **1.02** | 1.00 | 1.03 | **0.99** | 0.98 | 1.01 | **0.99** | 0.94 | 1.03 | |
| Inclusiveness | **0.97** | 0.95 | 0.99 | **0.99** | 0.97 | 1.00 | **0.99** | 0.97 | 1.01 | **0.95** | 0.90 | 0.99 | |
| Influence on schedule | **0.96** | 0.94 | 0.99 | **0.96** | 0.94 | 0.97 | **0.97** | 0.95 | 1.00 | **0.95** | 0.90 | 1.01 | |
| Influence on work | **0.93** | 0.91 | 0.95 | **0.91** | 0.89 | 0.92 | **0.96** | 0.94 | 0.98 | **0.94** | 0.90 | 0.99 | |
| Justice | **0.94** | 0.92 | 0.96 | **0.95** | 0.93 | 0.96 | **0.97** | 0.95 | 0.98 | **0.93** | 0.90 | 0.97 | |
| Leadership quality | **0.98** | 0.96 | 1.00 | **0.99** | 0.98 | 1.01 | **0.98** | 0.96 | 1.00 | **0.98** | 0.94 | 1.03 | |
| Predictability | **0.96** | 0.94 | 0.97 | **0.97** | 0.96 | 0.98 | **0.96** | 0.94 | 0.98 | **0.95** | 0.91 | 1.00 | |
| Role clarity | **0.96** | 0.93 | 0.98 | **0.98** | 0.95 | 1.00 | **0.96** | 0.92 | 0.99 | **0.96** | 0.90 | 1.03 | |
| Sexual harassment, last 12 months | **0.94** | 0.90 | 0.98 | **0.95** | 0.92 | 0.98 | **0.92** | 0.88 | 0.97 | **0.98** | 0.89 | 1.06 | |
| Skill discretion | **0.91** | 0.89 | 0.93 | **0.92** | 0.91 | 0.94 | **0.93** | 0.91 | 0.96 | **0.90** | 0.86 | 0.95 | |
| Threats, last 12 months | **0.93** | 0.91 | 0.96 | **0.97** | 0.95 | 0.99 | **0.91** | 0.88 | 0.94 | **0.96** | 0.91 | 1.02 | |
| Trust | **0.96** | 0.94 | 0.99 | **0.97** | 0.96 | 0.99 | **0.99** | 0.97 | 1.02 | **0.95** | 0.90 | 1.00 | |
| Violence, last 12 months | **0.96** | 0.93 | 0.99 | **0.94** | 0.92 | 0.97 | **0.91** | 0.88 | 0.95 | **1.04** | 0.97 | 1.12 | |
| Work demands | **0.97** | 0.95 | 0.99 | **1.02** | 1.00 | 1.04 | **0.99** | 0.97 | 1.01 | **0.95** | 0.90 | 0.99 | |
| **Cognitive and emotional reactions** | | | | | | | | | | | | | |
| Job satisfaction | **0.93** | 0.91 | 0.94 | **0.95** | 0.94 | 0.96 | **0.95** | 0.93 | 0.97 | **0.92** | 0.88 | 0.96 | |
| Perceived stress, last 6 months | **0.89** | 0.87 | 0.92 | **0.92** | 0.90 | 0.93 | **0.92** | 0.89 | 0.94 | **0.91** | 0.86 | 0.96 | |

Estimates of *All improvements* and *psychosocial working conditions* are adjusted for: Age, sex, occupational group, household income, marital status, part-time/full-time employment, seniority and workplace. Estimates of *all cognitive and emotional reactions* are adjusted for the above mentioned factors and also *psychosocial working conditions.*

**Table S6:** Rate ratios and 95% confidence intervals of sickness absence of different lengths in etiologic contrast scenarios for **men**. N=5316

|  | Total sickness  absence | | | Short-term  sickness  absence | | | Intermediate-term sickness  absence | | | Long-term  sickness  absence | | |  |
| --- | --- | --- | --- | --- | --- | --- | --- | --- | --- | --- | --- | --- | --- |
|  | RR | 95% CI,  lower | 95% CI,  higher | RR | 95% CI,  lower | 95% CI,  higher | RR | 95% CI,  lower | 95% CI,  higher | RR | 95% CI,  lower | 95% CI,  higher | |
| All improvements | **0.48** | 0.42 | 0.54 | **0.57** | 0.53 | 0.61 | **0.56** | 0.50 | 0.63 | **0.50** | 0.36 | 0.64 | |
| **Psychosocial working conditions** | | | | | | | | | | | | | |
| Bullying, last 12 months | **0.88** | 0.85 | 0.91 | **0.94** | 0.91 | 0.96 | **0.88** | 0.85 | 0.91 | **0.87** | 0.81 | 0.93 | |
| Collaboration | **0.99** | 0.97 | 1.01 | **1.01** | 0.99 | 1.02 | **0.99** | 0.97 | 1.01 | **0.98** | 0.94 | 1.02 | |
| Inclusiveness | **0.96** | 0.94 | 0.98 | **0.98** | 0.96 | 0.99 | **0.99** | 0.97 | 1.01 | **0.95** | 0.91 | 0.99 | |
| Influence on schedule | **0.97** | 0.94 | 0.99 | **0.95** | 0.94 | 0.97 | **0.97** | 0.95 | 0.99 | **0.97** | 0.92 | 1.01 | |
| Influence on work | **0.94** | 0.92 | 0.96 | **0.91** | 0.90 | 0.93 | **0.96** | 0.94 | 0.98 | **0.95** | 0.90 | 0.99 | |
| Justice | **0.94** | 0.93 | 0.96 | **0.94** | 0.93 | 0.95 | **0.97** | 0.95 | 0.98 | **0.94** | 0.91 | 0.97 | |
| Leadership quality | **0.97** | 0.96 | 0.99 | **0.98** | 0.97 | 1.00 | **0.99** | 0.97 | 1.00 | **0.98** | 0.94 | 1.02 | |
| Predictability | **0.95** | 0.93 | 0.97 | **0.96** | 0.95 | 0.97 | **0.96** | 0.94 | 0.97 | **0.96** | 0.92 | 0.99 | |
| Role clarity | **0.96** | 0.93 | 0.99 | **0.97** | 0.95 | 0.99 | **0.96** | 0.93 | 0.99 | **0.97** | 0.91 | 1.02 | |
| Sexual harassment, last 12 months | **0.95** | 0.92 | 0.98 | **0.96** | 0.93 | 0.99 | **0.93** | 0.89 | 0.98 | **0.99** | 0.91 | 1.07 | |
| Skill discretion | **0.92** | 0.89 | 0.94 | **0.92** | 0.90 | 0.93 | **0.94** | 0.91 | 0.96 | **0.91** | 0.87 | 0.96 | |
| Threats, last 12 months | **0.95** | 0.92 | 0.97 | **0.98** | 0.96 | 1.00 | **0.92** | 0.89 | 0.95 | **0.97** | 0.91 | 1.03 | |
| Trust | **0.96** | 0.94 | 0.98 | **0.96** | 0.95 | 0.98 | **0.99** | 0.97 | 1.01 | **0.95** | 0.90 | 0.99 | |
| Violence, last 12 months | **0.97** | 0.94 | 1.00 | **0.95** | 0.93 | 0.98 | **0.93** | 0.90 | 0.96 | **1.04** | 0.97 | 1.11 | |
| Work demands | **0.97** | 0.96 | 0.99 | **1.01** | 0.99 | 1.02 | **0.99** | 0.97 | 1.01 | **0.95** | 0.91 | 0.99 | |
| **Cognitive and emotional reactions** | | | | | | | | | | | | | |
| Job satisfaction | **0.93** | 0.91 | 0.95 | **0.94** | 0.93 | 0.95 | **0.95** | 0.93 | 0.97 | **0.93** | 0.89 | 0.96 | |
| Perceived stress, last 6 months | **0.90** | 0.88 | 0.93 | **0.92** | 0.90 | 0.93 | **0.93** | 0.90 | 0.95 | **0.92** | 0.87 | 0.96 | |

Estimates of *All improvements* and *psychosocial working conditions* are adjusted for: Age, sex, occupational group, household income, marital status, part-time/full-time employment, seniority and workplace. Estimates of *all cognitive and emotional reactions* are adjusted for the above mentioned factors and also *psychosocial working conditions.*

**Table S7:** Rate ratios and 95% confidence intervals of total sickness absence within occupational groups. N=24 990

|  | Total sickness absence | | | | | |
| --- | --- | --- | --- | --- | --- | --- |
|  | Etiologic scenarios | | | Realistic scenarios | | |
|  | RR | 95% CI,  lower | 95% CI,  higher | RR | 95% CI,  lower | 95% CI,  higher |
| Overall | **0,46** | 0,40 | 0,52 | **0,70** | 0,64 | 0,76 |
| Physicians | **0,46** | 0,40 | 0,52 | **0,73** | 0,68 | 0,79 |
| Nurses | **0,46** | 0,40 | 0,52 | **0,70** | 0,64 | 0,75 |
| Social and health care employees | **0,49** | 0,42 | 0,55 | **0,66** | 0,60 | 0,73 |
| Other health care employees^c^ | **0,47** | 0,41 | 0,54 | **0,72** | 0,66 | 0,78 |
| Pedagogical employees | **0,54** | 0,45 | 0,63 | **0,69** | 0,61 | 0,78 |
| Service and technical employees | **0,49** | 0,42 | 0,56 | **0,73** | 0,66 | 0,80 |
| Administrative leaders | **0,42** | 0,35 | 0,48 | **0,82** | 0,75 | 0,89 |
| Administrative employees | **0,44** | 0,37 | 0,50 | **0,68** | 0,62 | 0,74 |

Estimates of *All improvements* are adjusted for: Age, sex, occupational group, household income, marital status, part-time/full-time employment, seniority and workplace.

**Table S8:** Rate ratios and 95% confidence intervals of sickness absence of different lengths in etiologic contrast scenarios adjusted for short-term, intermediate-term and long-term sickness in the year before baseline. N=24 990

|  | Total sickness  absence | | | Short-term  sickness  absence | | | Intermediate-term sickness  absence | | | Long-term  sickness  absence | | |  |
| --- | --- | --- | --- | --- | --- | --- | --- | --- | --- | --- | --- | --- | --- |
|  | RR | 95% CI,  lower | 95% CI,  higher | RR | 95% CI,  lower | 95% CI,  higher | RR | 95% CI,  lower | 95% CI,  higher | RR | 95% CI,  lower | 95%CI,  higher | |
| All improvements | **0.48** | 0.44 | 0.53 | **0.67** | 0.62 | 0.71 | **0.59** | 0.52 | 0.65 | **0.44** | 0.33 | 0.56 | |
| **Psychosocial working conditions** | | | | | | | | | | | | | |
| Bullying. last 12 months | **0.85** | 0.82 | 0.88 | **0.92** | 0.90 | 0.95 | **0.86** | 0.82 | 0.89 | **0.82** | 0.76 | 0.88 | |
| Collaboration | **1.00** | 0.98 | 1.03 | **1.02** | 1.01 | 1.04 | **1.00** | 0.98 | 1.03 | **1.00** | 0.95 | 1.04 | |
| Inclusiveness | **0.97** | 0.95 | 0.99 | **0.99** | 0.98 | 1.01 | **1.00** | 0.97 | 1.03 | **0.95** | 0.91 | 0.99 | |
| Influence on schedule | **0.97** | 0.95 | 1.00 | **0.96** | 0.94 | 0.98 | **0.98** | 0.95 | 1.01 | **0.98** | 0.93 | 1.03 | |
| Influence on work | **0.95** | 0.93 | 0.98 | **0.91** | 0.90 | 0.93 | **0.97** | 0.95 | 1.00 | **0.97** | 0.91 | 1.02 | |
| Justice | **0.95** | 0.93 | 0.97 | **0.95** | 0.93 | 0.97 | **0.97** | 0.95 | 0.99 | **0.94** | 0.90 | 0.98 | |
| Leadership quality | **0.99** | 0.96 | 1.01 | **1.00** | 0.99 | 1.02 | **0.99** | 0.97 | 1.01 | **0.98** | 0.94 | 1.03 | |
| Predictability | **0.96** | 0.94 | 0.98 | **0.98** | 0.96 | 0.99 | **0.97** | 0.95 | 0.99 | **0.96** | 0.91 | 1.01 | |
| Role clarity | **0.96** | 0.93 | 0.99 | **0.99** | 0.97 | 1.02 | **0.95** | 0.92 | 0.99 | **0.97** | 0.91 | 1.03 | |
| Sexual harassment. last 12 months | **0.96** | 0.92 | 1.00 | **0.97** | 0.94 | 1.00 | **0.94** | 0.89 | 0.99 | **1.01** | 0.93 | 1.10 | |
| Skill discretion | **0.92** | 0.89 | 0.94 | **0.93** | 0.92 | 0.95 | **0.95** | 0.92 | 0.97 | **0.91** | 0.87 | 0.95 | |
| Threats. last 12 months | **0.93** | 0.90 | 0.96 | **0.97** | 0.95 | 1.00 | **0.90** | 0.87 | 0.93 | **0.95** | 0.89 | 1.02 | |
| Trust | **0.97** | 0.94 | 0.99 | **0.98** | 0.96 | 0.99 | **1.01** | 0.98 | 1.03 | **0.95** | 0.90 | 1.00 | |
| Violence. last 12 months | **0.97** | 0.94 | 1.00 | **0.97** | 0.94 | 0.99 | **0.93** | 0.90 | 0.97 | **1.02** | 0.95 | 1.10 | |
| Work demands | **0.97** | 0.95 | 1.00 | **1.03** | 1.01 | 1.05 | **0.99** | 0.97 | 1.02 | **0.95** | 0.90 | 0.99 | |
| **Cognitive and emotional reactions** | | | | | | | | | | | | | |
| Job satisfaction | **0.93** | 0.91 | 0.95 | **0.95** | 0.94 | 0.97 | **0.95** | 0.93 | 0.97 | **0.92** | 0.88 | 0.95 | |
| Perceived stress. last 6 months | **0.89** | 0.86 | 0.91 | **0.92** | 0.90 | 0.93 | **0.91** | 0.89 | 0.94 | **0.90** | 0.86 | 0.95 | |

Estimates of *All improvements* and *psychosocial working conditions* are adjusted for: Age, sex, occupational group, household income, marital status, part-time/full-time employment, seniority and workplace. Estimates of *all cognitive and emotional reactions* are adjusted for the above mentioned factors and also *psychosocial working conditions.*

**Table S9:** Rate ratios and 95% confidence intervals of sickness absence of different lengths in etiologic contrast scenarios weighted by inverse probability weights to account for conditioning on prior and future events. N=24 990

|  | Short-term  sickness  absence^a^ | | | | Intermediate-term sickness  absence^b^ | | | | | | | Long-term  sickness  absence^c^ | | | | |  |
| --- | --- | --- | --- | --- | --- | --- | --- | --- | --- | --- | --- | --- | --- | --- | --- | --- | --- |
|  | RR | 95% CI,  lower | 95% CI,  higher | | RR | | | 95% CI,  lower | | 95% CI,  higher | | RR | | 95% CI,  lower | | 95%CI,  higher | |
| All improvements | **0.60** | 0.55 | 0.65 | | **0.55** | | | 0.48 | | 0.61 | | **0.48** | | 0.36 | | 0.60 | |
| **Psychosocial working conditions** | | | | | | | | | | | | | | | | | |
| Bullying, last 12 months | **0.92** | 0.90 | | 0.94 | | **0.86** | | 0.83 | | | 0.90 | | **0.85** | | 0.78 | 0.92 | |
| Collaboration | **1.01** | 0.99 | | 1.02 | | **0.99** | | 0.97 | | | 1.00 | | **0.98** | | 0.94 | 1.03 | |
| Inclusiveness | **0.98** | 0.97 | | 1.00 | | **0.99** | | 0.96 | | | 1.01 | | **0.94** | | 0.90 | 0.99 | |
| Influence on schedule | **0.96** | 0.94 | | 0.97 | | **0.97** | | 0.94 | | | 1.00 | | **0.95** | | 0.89 | 1.01 | |
| Influence on work | **0.91** | 0.89 | | 0.92 | | **0.96** | | 0.94 | | | 0.98 | | **0.95** | | 0.90 | 1.00 | |
| Justice | **0.95** | 0.94 | | 0.97 | | **0.96** | | 0.95 | | | 0.98 | | **0.93** | | 0.90 | 0.97 | |
| Leadership quality | **0.99** | 0.98 | | 1.00 | | **0.98** | | 0.96 | | | 1.00 | | **0.98** | | 0.94 | 1.02 | |
| Predictability | **0.97** | 0.95 | | 0.98 | | **0.96** | | 0.94 | | | 0.98 | | **0.95** | | 0.91 | 0.99 | |
| Role clarity | **0.98** | 0.96 | | 1.00 | | **0.96** | | 0.93 | | | 0.99 | | **0.96** | | 0.89 | 1.02 | |
| Sexual harassment, last 12 months | **0.96** | 0.93 | | 0.99 | | **0.92** | | 0.88 | | | 0.97 | | **1.00** | | 0.91 | 1.09 | |
| Skill discretion | **0.93** | 0.91 | | 0.94 | | **0.94** | | 0.91 | | | 0.96 | | **0.91** | | 0.86 | 0.97 | |
| Threats, last 12 months | **0.97** | 0.95 | | 0.99 | | **0.92** | | 0.89 | | | 0.94 | | **0.96** | | 0.91 | 1.01 | |
| Trust | **0.97** | 0.95 | | 0.98 | | **0.99** | | 0.97 | | | 1.01 | | **0.96** | | 0.91 | 1.02 | |
| Violence, last 12 months | **0.94** | 0.92 | | 0.97 | | **0.92** | | 0.89 | | | 0.95 | | **1.04** | | 0.97 | 1.11 | |
| Work demands | **1.01** | 1.00 | | 1.03 | | **0.99** | | 0.97 | | | 1.01 | | **0.94** | | 0.90 | 0.98 | |
| **Cognitive and emotional reactions** | | | | | | | | | | | | | | | | | |
| Job satisfaction | **0.95** | 0.94 | | 0.96 | | | **0.95** | | 0.94 | | 0.97 | | **0.91** | | 0.87 | 0.95 |  |
| Perceived stress. last 6 months | **0.92** | 0.90 | | 0.94 | | | **0.93** | | 0.91 | | 0.95 | | **0.91** | | 0.86 | 0.96 |  |

Estimates of *All improvements* and *psychosocial working conditions* are adjusted for: Age, sex, occupational group, household income, marital status, part-time/full-time employment, seniority and workplace. Estimates of *all cognitive and emotional reactions* are adjusted for the above mentioned factors and also *psychosocial working conditions.*

1. Weighted by inverse probability of no intermediate-term or long term sickness absence
2. Weighted by inverse probability of no short term or long term sickness absence
3. Weighted by inverse probability of no short term or intermediate-term sickness absence
